# Are changes in glycaemic control associated with diabetes-specific quality of life and health status in screen-detected type 2 diabetes patients? Four-year follow up of the ADDITION-Cambridge cohort

**DOI:** 10.1002/dmrr.2559

**Published:** 2015-01-15

**Authors:** L Kuznetsov, G H Long, S J Griffin, R K Simmons

**Affiliations:** 1 MRC Epidemiology Unit, University of Cambridge School of Clinical Medicine, Institute of Metabolic ScienceCambridge, UK; 2 The Primary Care Unit, Institute of Public Health, University of CambridgeCambridge, UK

**Keywords:** HbA_1c_, diabetes-specific quality of life, ADDQoL, health-status, SF-36, type 2 diabetes

## Abstract

**Background:**

Interventions that improve HbA_1c_ levels do not necessarily improve health-related quality of life (QoL). This issue may be particularly relevant in asymptomatic diabetes patients detected earlier in the course of the disease.

**Methods:**

HbA_1c_, diabetes-specific QoL (ADDQoL) and health status were measured in 510 screen-detected diabetes patients from the ADDITION-Cambridge trial at 1 and 5 years post diagnosis. Multivariable logistic/linear regression was used to quantify the longitudinal association between change in HbA_1c_ from 1 to 5 years and ADDQoL and health status at 5 years, adjusting for age, sex, education and trial group; alcohol consumption, smoking, physical activity, plasma vitamin C, HbA_1c_, ADDQoL or health status at 1 year, and glucose-lowering medication at 5 years.

**Results:**

From 1 to 5 years, median HbA_1c_ interquartile range increased from 6.3% (5.9–6.8) to 6.8% (6.4–7.4); the median ADDQoL score and mean health status physical health summary score decreased from -0.4 (-1 to -0.08) to -0.5 (-1.08 to -0.09) (suggesting an adverse impact of diabetes on QoL) and by -0.79 (8.94) points, respectively. Increases in HbA_1c_ were independently associated with reporting a negative impact of diabetes on QoL (OR = 1.38, 95% CI: 1.03 to 1.85) but not with the health status summary scores.

**Conclusions:**

Increases in HbA_1c_ from 1 to 5 years post-diagnosis were independently associated with increased odds of reporting a negative impact of diabetes on QoL. While our results suggest that efforts to reduce HbA_1c_ do not adversely affect health-related QoL, large numbers of participants still report a negative impact of diabetes on their QoL 5 years post-diagnosis. © 2014 The Authors. *Diabetes/Metabolism Research and Reviews* published by John Wiley & Sons, Ltd.

## Introduction

Among individuals with diabetes, control of cardiovascular risk factors, including blood glucose, is achieved by promotion of healthy lifestyle behaviours and an often complex medication regimen. Treatment regimens may impact on a patient's illness experience and their health-related quality of life (QoL), which is an important outcome indicator in diabetes care [1]. Interventions that improve HbA_1c_ levels do not necessarily improve health-related QoL [2]. This issue may be particularly relevant in asymptomatic patients detected earlier in the course of the disease, where the burden of treatment may be higher than the burden of the disease. The number of individuals with early or screen-detected diabetes is expected to grow with the advent of national screening programmes [3] and there are outstanding uncertainties about how to treat this group. Recent concerns about intensive treatment of glucose [4] also highlight the importance of considering the relationship between glycaemic control and health-related QoL when developing treatment guidelines for screen-detected patients.

Previous studies assessing the longitudinal relationship between HbA_1c_ and health-related QoL have focused on individuals with established diabetes (mean diabetes duration 8.7 to 11.2 years) and have been of short duration (maximum follow-up of 12 months). Some studies report that improvements in HbA_1c_ are associated with higher levels of diabetes control [5], emotional well-being [6], reduced diabetes symptoms [6,7], and increased treatment satisfaction [7], while others find no longitudinal association between HbA_1c_ and diabetes-specific QoL [5] or health status [7,8]. Studies with longer follow-up [6] and larger samples [8] are needed to examine the relationship between HbA_1c_ and health-related QoL, particularly in screen-detected individuals, as few studies focus on this high-risk group.

A cross-sectional examination of the ADDITION-Europe trial cohort, encompassing 3057 individuals with screen-detected diabetes, demonstrated that diabetes-specific QoL was independently associated with HbA_1c_ at 5 years post-diagnosis [9]. We extend this work by exploring the prospective association between QoL and HbA_1c_ using data from the Cambridge arm of the ADDITION-Europe trial. We (i) examined change in HbA_1c_ and diabetes-specific QoL and health status from 1- to 5-year follow-up and (ii) quantified the association between change in HbA_1c_ and diabetes-specific QoL and health status at 5 years of follow-up.

## Materials and methods

The study design has been described elsewhere [10]. In brief, ADDITION-Cambridge consisted of two phases: a screening phase and a pragmatic, cluster-randomized, parallel-group trial. Individuals were eligible for invitation to the screening phase of the study if they were aged 40–69 years, registered with one of the 49 participating general practice surgeries based in the Eastern region of England, not known to have diabetes, and had a diabetes risk score corresponding to the top 25% of the population distribution [11]. Exclusion criteria included pregnancy, lactation, a psychiatric illness that might invalidate informed consent, or an illness with a likely prognosis of less than 1 year. In total, 33 539 eligible individuals were invited to take part in the screening programme. Of these, 867 individuals were found to have diabetes, according to World Health Organization criteria [12], and agreed to participate in the treatment trial. All participants gave written informed consent and the study was approved by the Eastern Multi-Regional Ethics Committee. ADDITION-Cambridge is registered as ISRCTN86769081.

### Measures

Baseline, 1-year and 5-year health assessments of ADDITION-Cambridge participants included physiological and anthropometric measures by trained staff following standard operating procedures. Standardized self-report questionnaires were used to collect information on age, sex, ethnicity, age when completed full-time education, lifestyle behaviours (smoking status, alcohol consumption), and prescribed medication. Self-reported physical activity was assessed using the validated EPAQ2 questionnaire [13] and expressed as total metabolic equivalent task (MET hours/week). Fruit and vegetable intake was assessed by measurement of levels of the biomarker plasma vitamin C, using a Fluoroskan Ascent FL fluorometer [14]. HbA_1c_ was analysed by ion-exchange high-performance liquid chromatography (Tosoh Bioscience, Redditch, UK).

Diabetes-specific QoL was assessed using the Audit of Diabetes Dependent Quality of Life (ADDQoL) [15], which measures an individual's perception and importance of the impact of diabetes on various aspects of their QoL [16]. Two different versions of the ADDQoL were used in ADDITION-Cambridge: version 18 at 1-year follow-up and version 19 at 5-year follow-up. We have included only the items identical at both time points (13 out of 18 and 19 items in the ADDQoL18 and ADDQoL19, respectively). Excluded items were: ‘holidays/leisure activities’, ‘travel/journeys’, ‘society/people reaction’, ‘dependence’, ‘enjoyment of food’, and ‘closest personal relationship’. Respondents rate the impact of diabetes on different domains on a scale from -3 (maximum negative impact) to +1/+3 (maximum positive impact), and then rate the importance of the domain for their QoL on a scale from 3 (very important) to 0 (not at all important) [17]. The weighted impact score for each domain was assessed by multiplying the unweighted rating by the importance rating, and ranges from -9 (maximum negative impact) to +3/+9 (maximum positive impact) [16]. To calculate an overall continuous Average Weighted Impact (AWI) score, the weighted ratings of applicable domains were summed and divided by the number of applicable domains [17]. A low continuous ADDQoL score reflects a negative impact of diabetes on QoL. The Cronbach's alpha of the ADDQoL unweighted items at 1- and 5-year follow-up was 0.83 and 0.84, respectively.

Health status was assessed using the 36-item Short Form Health Survey (SF-36) [18]. This self-reported measure consists of 36 items which form eight subscales: physical functioning, role—physical, bodily pain, role—emotional, general health, vitality, social functioning, role—emotional, and mental health ranging from 0 to 100, with higher scores indicating better health [18]. For this analysis two summary measures (the Physical Component Summary (PCS) and the Mental Component Summary (MCS) scores) were computed and examined. Cronbach's alpha indicated satisfactory reliability of all SF-36 health domain scales, ranging from 0.78 to 0.93 at 1 year and from 0.74 to 0.93 at 5 years. This analysis includes data taken from the 1- and 5-year health assessments (except sex and education measures taken at baseline).

### Statistical analysis

Only cases with complete data were included in the analysis (*n* = 510). Descriptive characteristics were summarized using means, standard deviations (SD), or frequencies. In order to examine differences between participants with and without complete data for analysis, we used the chi-square test, the t-test, the Mann-Whitney U and Wilcoxon signed rank test where appropriate. Change in HbA_1c_ from 1- to 5-year follow-up was calculated by subtracting 1-year values from 5-year follow-up values. Due to the highly skewed distribution of the continuous ADDQoL score, a binary ADDQoL variable was generated, which categorized patients into those reporting a negative (‘1’), or no/positive impact of diabetes (‘0’); the range of the continuous ADDQoL score values at 1 year was -9 to -0.07 (‘1’) and 0 to 1.15 (‘0’), and -7.36 to -0.07 (‘1’) and 0 to 0.23 (‘0’) at 5 years. Multivariable logistic and linear regression was performed to quantify the association between change in HbA_1c_ (independent variable) over 4 years and the binary ADDQoL variable (dependent variable), and the SF-36 summary scores (dependent variable) measured at the 5-year health assessment. Model 1 was adjusted for the *a priori* confounders sex and age at completion of full-time education (<16 years or ≥16 years of age), 1-year values for age, HbA_1c_, the binary ADDQoL variable or the SF-36 summary scores, and trial group. Model 2 was additionally adjusted for the number of glucose-lowering medications at 5-year follow-up to capture the intensity of glucose lowering treatment regimen. To adjust for health behaviours, Model 3 included alcohol consumption (those who meet the guidelines on alcohol consumption and those who do not (men: ≤21 units/week or >21 units/week; women: ≤14 units/week or >14 units/week) [19]), smoking (non-smoker/ex-smoker or current smoker), physical activity (total MET hours/week), and plasma vitamin C. Models were run separately by sex and trial group, and as results were similar, the data were pooled and presented for the whole cohort. The residuals of linear regression models were examined to ensure that they were approximately normally distributed. All logistic and linear regression results are presented as odds ratios (OR) or unstandardized b-coefficients with their 95% confidence intervals. Statistical significance was set at *p* < 0.05. Statistical analyses were performed using SPSS for Windows 19.0 (SPSS, Inc., Chicago, IL) and Stata/SE 12.0 (Stata-Corp, College Station, TX).

## Results

Of those still alive, 736/860 (86%) ADDITION-Cambridge participants returned for their 1-year follow-up health assessment, and 653/812 (80%) for their 5-year health assessment.

There were no significant differences between participants included in the analysis (*n* = 510) and those who were not included because of missing data (*n* = 301) for sex, 1-year alcohol consumption, smoking status and change in HbA_1c_. However, those who were not included were more likely to be older, to have completed their education at younger age, to have lower mean plasma vitamin C, and to be less physically active compared to those who were included.

At the 1-year health assessment, the mean age (SD) of participants was 61.8 (7.1) years, 62% were male, 59% of the cohort were obese (BMI ≥30 kg/m^2^), and the majority of participants were not taking any glucose-lowering medication (69%) (Table1). After 4 years, the median (interquartile range, IQR) HbA_1c_ increased from 6.3% (5.9 to 6.8) to 6.8% (6.4 to 7.4), *p* < 0.001 (Table2). Almost half (46%) the participants were taking at least one glucose-lowering medication and 15% two glucose-lowering medications; the median (range) of glucose-lowering medication was 1 (0 to 3) (data not shown).

**Table 1:** Participants characteristics at 1 year in the ADDITION-Cambridge cohort (*n* = 510)

Characteristics	Mean (SD)
Age (years)	61.8 (7.14)
Male sex^a^, % (*n*)	62.0 (316)
Full-time education completed at ≥16 years^a^, % (*n*)	56.5 (288)
Caucasian ethnicity^a^, % (*n*)	97.8 (499)
Body mass index (kg/m^2^)	
Men	31.4 (5.20)
Women	32.9 (5.92)
Number of glucose-lowering medications, % (*n*)	
0	69.2 (353)
1	27.3 (139)
2	3.3 (17)
3	0.2 (1)
Number of glucose-lowering medication, median (range)	0 (0 to 3)
Smoking status (current smoker), % (*n*)	12.7 (65)
Alcohol consumption (those who meet the guidelines on alcohol consumption), % (*n*)	91.6 (467)
Physical activity (MET hours/week)	12.2 (7.2)
Plasma vitamin C, mg/dL	56.7 (24.0)

Values are means (SD) unless stated otherwise.

a Measured at baseline.

**Table 2:** Change in HbA_1c_, diabetes-specific quality of life and general health status between 1 and 5 years in the ADDITION-Cambridge cohort (*n* = 510)

Variable	1 year	5 years	Absolute change (95% CI)
HbA_1c_ (%)	6.5 (0.86)	7.0 (0.99)	0.54 (0.45 to 0.63)
HbA_1c_ (%), median (IQR)	6.3 (5.9 to 6.8)	6.8 (6.4 to 7.4)	–
Continuous ADDQoL score	-0.7 (1.12)	-0.9 (1.24)	-0.13 (-0.22 to -0.03)
Continuous ADDQoL score, median (IQR)	-0.4 (-1.0 to -0.08)	-0.5 (-1.08 to -0.09)	–
Participants reporting a negative impact of diabetes (the binary ADDQoL variable < 0), % (*n*)	76.5 (390)	80.8 (412)	–
SF-36 physical health summary score	45.6 (10.87)	44.8 (11.25)	-0.79 (-1.57 to -0.01)
SF-36 mental health summary score	52.7 (9.28)	54.2 (8.23)	1.50 (0.74 to 2.26)

Values are means (SD) unless stated otherwise.

HbA_1c_, glycosylated haemoglobin.

ADDQoL AWI score, the overall average weighted impact score of the ADDQoL (range -9 to +3).

IQR, interquartile range.

Between 1 and 5 years, the median (IQR) continuous ADDQoL score decreased from -0.4 (-1 to -0.08) to -0.5 (-1.08 to -0.09), *p* = 0.027. The proportion of participants reporting a negative impact of diabetes on their QoL increased from 76.5% to 81% (*p* < 0.001) and the mean SF-36 PCS score decreased from 45.6 (10.87) to 44.8 (11.25) (*p* = 0.047), suggesting an adverse impact of diabetes on QoL. The mean MCS score increased from 52.7 (9.28) to 54.2 (8.2) (*p* < 0.001) (Table2).

When adjusted for sex, education, trial group and 1-year values for age, HbA_1c_, the binary ADDQoL variable (Model 1), change in HbA_1c_ was independently associated with the binary ADDQoL variable at 5-year follow-up (OR = 1.39, 95% CI: [1.05, 1.86]) (Table3). After further adjustment for number of glucose-lowering medications at 5 years (OR = 1.35 [1.01 to 1.79]) (Model 2) and diet and physical activity at 1 year (Model 3), the association remained significant (OR = 1.38 [1.03, 1.85]) suggesting that every one percentage-point increase in HbA_1c_ was associated with a 38% increase in reporting a negative impact of diabetes on QoL, respectively. Neither of the SF-36 summary scores were associated with change in HbA_1c_ (PCS: b-coefficient = 0.1 [-0.72, 0.91]; MCS: b-coefficient = -0.05 [-0.74, 0.64]) (Table3).

**Table:** Association between change in HbA_1c_ from 1 to 5 years and diabetes-specific quality of life and general health status at 5 years in the ADDITION-Cambridge cohort (*n* = 510)

Variables	Estimates
Binary ADDQoL variable (no impact/positive impact of diabetes on QoL = 0)	OR (95% CI)^a^
Model 1	1.39 (1.05 to 1.86)*
Model 2	1.35 (1.01 to 1.79)*
Model 3	1.38 (1.03 to 1.85)*
SF-36 physical health summary score (continuous)	b-coefficient (95% CI)
Model 1	0.1 (-0.70 to 0.89)
Model 2	0.09 (-0.71 to 0.90)
Model 3	0.10 (-0.72 to 0.91)
SF-36 mental health summary score (continuous)	b-coefficient (95% CI)
Model 1	-0.17 (-0.84 to 0.50)
Model 2	-0.04 (-0.72 to 0.64)
Model 3	-0.05 (-0.74 to 0.64)

Model 1: adjusted for sex and education, 1-year values for age, HbA_1c_ and the binary ADDQoL variable or SF-36 summary scores, and trial group.

Model 2: as for Model 1 and additionally adjusted for number of glucose-lowering medication at 5 years.

Model 3: as for Model 2 and additionally adjusted for 1-year alcohol consumption, smoking, physical activity, and plasma vitamin C.

* *p* < 0.05.

a aEvery one percentage-point increase in HbA_1c_ is associated with increased odds of reporting a negative impact of diabetes on QoL.

## Discussion

Increases in HbA_1c_ from 1 to 5 years post-diagnosis were independently associated with increased odds of reporting a negative impact of diabetes on QoL in screen-detected diabetes patients. This association remained significant after adjustment for glucose-lowering medication and lifestyle behaviours. In contrast, the SF-36 physical and mental health summary scores were not associated with change in HbA_1c_ over the same time period. Our results suggest that efforts to reduce glycaemia in the 5 years following diagnosis do not adversely affect diabetes-specific QoL. However, large numbers of participants still report a negative impact of diabetes on their QoL 5 years post-diagnosis.

There was only a small change over the 4-year period in diabetes-specific QoL and general health status in our cohort. A prospective observational study among type 1 and type 2 (40%) insulin-requiring individuals with diabetes (mean age 47 years, diabetes duration 15 years) attending an intensive diabetes self-management program in Australia reported a small increase in the ADDQoL of 1.9 and 3.9 points at 4 and 12 months, respectively [20]. In the ADDITION-Cambridge cohort the change in the continuous ADDQoL score was -0.13 (1.12) over 4 years. This comparison should be interpreted with caution due the different study populations and different versions of the ADDQoL used in each study. The change in mean SF-36 PCS (-0.79 (8.9)) and MCS (1.5 (8.8)) scores between 1 and 5 years in the ADDITION-Cambridge cohort is similar to that reported in previous studies. For example, in a US cohort of individuals (mean age 56 years) with type 1 and type 2 (84%) diabetes attending diabetes educational workshops, 1-year change in mean SF-36 PCS and MCS score were -1 and 3.8, respectively [21]. Comparisons should again be treated with caution because of the inclusion of both types of diabetes patients and shorter follow-up time. Furthermore, mean HbA_1c_ values decreased from 8.8% at baseline to 7.2% at 1 year in the US cohort, while HbA_1c_ levels increased from 6.5% to 7% in our cohort.

Our results are in broad agreement with previous studies reporting an inverse longitudinal association between HbA_1c_ and diabetes-specific QoL. For example, in a predominantly male (97%) US cohort of patients completing diabetes self-management programs (mean age 64 years) improvements in HbA_1c_ were associated with improved QoL on a diabetes control scale at 1-year follow-up [5]. In another US cohort (mean age 62 years, median duration of diabetes 10 years) better glycaemic control over 1 year was associated with reduced patient-reported diabetes symptoms, symptom distress and treatment satisfaction [7]. Our results expand on previous research by showing that diabetes-specific QoL is sensitive to glycaemic changes even in well-controlled diabetes patients early in the disease trajectory.

Some studies have suggested that a clinically meaningful difference for the SF-36 PCS and MCS scores is at least a 2.5 point change [22]. Given that the difference in the SF-36 PCS and MCS scores between 1- and 5-year follow-up in our cohort was only -0.79 and 1.5 points respectively, this suggests that there was little change in general health status over 4 years. This may be linked to the small increase in median HbA_1c_ (0.5%). Only increases in HbA_1c_ of 1% or greater have been associated with substantial decreases in health-related QoL [23]. It is therefore likely that diabetes and hyperglycaemia have limited impact on generic health measures early in the disease trajectory. Indeed, most previous studies do not find a significant association between HbA_1c_ and general health status in patients with established diabetes [7,8]. The lack of an association might be explained by the fact that our participants were early in the course of the disease, had good glycaemic control (only 11 participants were on insulin at 5-year follow-up) and their general health status might only be impaired when complications develop [24]. Further, the SF-36 is a generic measure which may have limited relevance when applied to a population with a specific disease such as diabetes [25]. It does not include some diabetes-relevant domains, for example, dietary restriction, which may significantly impair a diabetes patients’ QoL. Diabetes-specific instruments, such as the ADDQoL, are more sensitive to change and responsive to subgroup differences than a generic instrument [15]. Our use of both a disease-specific instrument and generic health status instrument allowed us to assess both diabetes-specific QoL and general health status in this patient group.

Tight glycaemic control among patients with longstanding disease may be associated with greater harms than benefits in the short term [4]. However, there is growing support for the long-term health benefits of tight blood glucose control early in the diabetes disease trajectory [26]. Our results suggest that newly diagnosed individuals may benefit from good glucose control through the adoption of healthier lifestyles and medical management. Some studies have reported that increasing treatment intensity in patients with diabetes is associated with worsening health-related QoL [27]. It is reassuring that despite increases in the number of oral glucose-lowering medication prescribed to each participant from 1 to 5 years, there were no adverse effects on diabetes-specific QoL and general health status in the ADDITION-Cambridge cohort. Adjusting for glucose-lowering medication did not change the point estimate or the significance of our results. Most ADDITION-Cambridge participants were prescribed metformin, which can cause initial side-effects but rarely causes weight gain or hypoglycaemia. Later in the disease trajectory, patients may be prescribed more medication leading to increased side-effects and the association between HbA_1c_ and QoL may therefore change.

The strengths of our study include its prospective design with 4 years of follow-up, the inclusion of both disease-specific QoL and generic health status instruments, and adjustment for a wide range of confounders. As expected, there were significant differences in some of the characteristics between those included and excluded from our analyses. We did not, however, find any differences between change in HbA_1c_ levels between those included and excluded. Nevertheless, as the participants excluded from the analysis were generally less healthy than those included, this may have led to an over-estimation of the association between change in HbA_1c_ and diabetes-specific QoL. The sample was largely Caucasian and middle-aged, which restricts generalisability to different populations. Self-reported lifestyle behaviours and medication intake may be subject to recall and social desirability bias. The use of fewer questions from the original ADDQoL questionnaire might have affected the instrument's sensitivity. However, the Cronbach's alpha indicated satisfactory reliability of the unweighted items in the shortened ADDQoL version at both time points. Our decision to dichotomize the continuous ADDQoL variable resulted in a loss of data and a potential reduction in statistical power. However, the skewed distribution of the ADDQoL variable at both time points proved impossible to transform due to a number of negative and zero values. Other groups have dichotomized the variable in similar ways [28,29].

In conclusion, even among well controlled diabetes patients early in the disease trajectory, increases in HbA_1c_ from 1 to 5 years post-diagnosis were independently associated with declines in diabetes-specific QoL. While our results suggest that efforts to reduce HbA_1c_ do not adversely affect health-related QoL, large numbers of participants still report a negative impact of diabetes on their QoL 5 years post-diagnosis.
